# VGLL4 Suppresses Malignant Phenotypes of Clear Cell Renal Cell Carcinoma Cells Through Modulation of Hippo/YAP and Wnt/β-Catenin Signaling Pathways

**DOI:** 10.3390/biomedicines14102334

**Published:** 2026-10-14

**Authors:** Yaru Zhang, Shuyao Wang, Zhipeng Zhao, Tao Yuan, Changyu Zhu, Lei Zhong

**Affiliations:** 1 Department of Pharmacy, Personalized Drug Research and Therapy Key Laboratory of Sichuan Province, Sichuan Provincial People’s Hospital, School of Medicine, University of Electronic Science and Technology of China, Chengdu 610072, China; zhangyaruro@163.com (Y.Z.); 202522130915@std.uestc.edu.cn (S.W.); luckyyuantao@163.com (T.Y.); 2 Department of Pharmacy, Evidence-Based Pharmacy Center, West China Second Hospital, Sichuan University, Chengdu 610041, China; zzp1996814txs@163.com; 3 Key Laboratory of Birth Defects and Related Diseases of Women and Children ( Sichuan University), Ministry of Education, Chengdu 610041, China; 4 State Key Laboratory of Neurology and Oncology Drug Development, Nanjing 210023, China

**Keywords:** VGLL4, ccRCC, YAP phosphorylation, dual regulation, prognostic indicator

## Abstract

**Background/Objectives:** Clear cell renal cell carcinoma (ccRCC), the most common and aggressive subtype of kidney cancer, continues to face challenges in early diagnosis and treatment, driving the need for reliable biomarkers and therapeutic targets. Vestigial-like family member 4 (VGLL4), a transcriptional cofactor, has been identified as a tumor suppressor in several malignancies. However, its clinical significance, biological functions, and underlying mechanisms in ccRCC remain largely unknown. **Methods:** In this study, we examined VGLL4 expression in ccRCC and normal tissues at both mRNA and protein levels and analyzed its correlation with clinicopathological features and patient prognosis. Functional experiments were performed to evaluate the effects of VGLL4 overexpression on ccRCC cell proliferation, migration, and invasion in vitro, as well as tumor growth in vivo. Integrated transcriptomic profiling and immunoblot validation were employed to explore the underlying signaling pathways. **Results:** We found that VGLL4 was downregulated in ccRCC tissues compared to normal counterparts at both mRNA and protein levels, and its low expression correlates with advanced disease stage and poorer overall survival. Functional experiments demonstrated that VGLL4 overexpression significantly suppressed the proliferation, migration, and invasion of ccRCC cells in vitro and effectively hindered tumor growth in vivo. Mechanistically, integrated transcriptomic profiling and immunoblot validation suggest that the tumor-suppressive effects of VGLL4 are mediated through the coordinated modulation of the Hippo/YAP and Wnt/β-catenin signaling pathways, at least in part by attenuating the activation and transcriptional activities of YAP and β-catenin. **Conclusions:** Collectively, these findings establish VGLL4 as a tumor suppressor in ccRCC and highlight its potential as a prognosis-related biomarker and candidate therapeutic target through its dual regulatory role in Hippo/YAP and Wnt/β-catenin signaling cascades.

## 1. Introduction

Renal cell carcinoma (RCC) is a prevalent malignant neoplasm of the urinary system, accounting for approximately 434,419 new cases and 155,702 deaths worldwide in 2022 [1]. Among its histological subtypes, clear cell RCC (ccRCC) represents the most common pathological type (~75%) and the most aggressive [2]. A major clinical challenge in ccRCC management is the frequent lack of early symptoms, leading to diagnosis at advanced or metastatic stages in a substantial proportion of patients. This delay in diagnosis severely compromises treatment efficacy and contributes to unfavorable prognoses [3,4]. Over the past two decades, advances in molecular targeted therapies, including VEGFR and mTOR inhibitors, as well as immunotherapies such as PD-1/PD-L1 checkpoint inhibitors, have improved survival in patients with advanced ccRCC. However, a substantial proportion of patients still experience disease progression due to intrinsic or acquired therapeutic resistance [5,6]. This persistent clinical challenge, combined with the heterogeneous molecular landscape of ccRCC, underscores the clinical need for discovering novel biomarkers and therapeutic targets to develop more effective treatment strategies.

The mammalian Vestigial-like family member 4 (VGLL4), a transcriptional cofactor homologous to the Drosophila vestigial gene, functions as a context-dependent regulator of development and tumorigenesis [7,8]. Unlike many transcriptional regulators, VGLL4 lacks a DNA-binding domain and instead modulates gene transcription through interaction with TEAD transcription factors via its two conserved Tondu (TDU) domains. Through this mechanism, VGLL4 antagonizes the pro-oncogenic YAP/TAZ–TEAD signaling axis, a central effector of the Hippo pathway [9]. Accumulating evidence highlights VGLL4’s tumor-suppressive roles and YAP’s tumor-promotive effects across malignancies, including gastric cancer, lung cancer, hepatocellular carcinoma, breast cancer and epidermal squamous cell carcinoma [8,10,11,12,13]. In these tumor types, elevated VGLL4 expression is generally associated with favorable patient prognosis, and either VGLL4 overexpression or treatment with VGLL4-mimicking peptides effectively suppresses cancer cell growth. However, emerging studies indicate that VGLL4 may exert divergent, context-dependent roles in certain malignancies. For example, in colorectal cancer, VGLL4 expression rises obviously in diseased tissues, with a marked increase from early to more advanced stages, while reduced VGLL4 expression prominently correlates with improved patient outcomes [14]. Additionally, VGLL4 has been implicated in promoting tumor immune evasion by enhancing PD-L1 expression [15]. Collectively, these findings underscore the tissue- and context-specific functions of VGLL4 and highlight the necessity for further investigation into its roles across different cancer types.

Given the dual and context-dependent role of VGLL4 across different malignancies, its function in ccRCC remains poorly defined and warrants systematic investigation. Whether VGLL4 acts predominantly as a tumor suppressor or exerts oncogenic effects within the specific microenvironment of ccRCC is a critical unresolved issue. Clarifying the expression patterns, clinical relevance, and molecular mechanisms of VGLL4 in ccRCC may provide valuable insights into its potential utility as a biomarker or therapeutic target. Accordingly, in this study, we aimed to comprehensively evaluate the clinical significance and biological functions of VGLL4 in ccRCC through integrated analysis of clinical samples, in vitro and in vivo experiments, as well as mechanistic studies. Our findings delineate the role of VGLL4 in ccRCC pathogenesis and shed light on its interaction with key oncogenic signaling pathways, thereby offering a rationale for future therapeutic strategies targeting VGLL4-associated regulatory networks.

## 2. Materials and Methods

### 2.1. Bioinformatics Analysis

VGLL4 mRNA expression levels across a pan-cancer spectrum were analyzed using the TIMER2 database (http://timer.cistrome.org/ accessed on 18 August 2026). Transcriptomic data and clinical information for ccRCC were downloaded from The Cancer Genome Atlas (TCGA) database (https://portal.gdc.cancer.gov/ accessed on 18 August 2026) and processed using R software version 4.3.1. The optimal cutoff for VGLL4 expression (TPM, primary tumor) was determined using X-tile 3.6.1. The association between VGLL4 expression and patient survival was evaluated using the “survminer” and “survival” R packages. Furthermore, VGLL4 protein expression data in ccRCC and normal kidney tissues were obtained from the UALCAN (https://ualcan.path.uab.edu/ accessed on 18 August 2026) and The Human Protein Atlas (HPA) (https://www.proteinatlas.org/ accessed on 18 August 2026) databases. Univariate and multivariate Cox regression analyses were performed to evaluate the association between VGLL4 expression and OS and DFS, with the multivariate model adjusted for age, sex, pathologic stage, and histologic grade.

### 2.2. Tissue Microarray (TMA)

A commercially available tissue microarray (TMA) (Product ID: HKidE060PG02, Shanghai Outdo Biotech Co., Shanghai, China), containing 30 pairs of ccRCC tissues and their matched adjacent non-cancerous renal tissues, was used in this study. Immunohistochemical (IHC) staining for VGLL4 (A18248, ABclonal, Woburn, MA, USA; 1:100) was performed by Outdo Biotech Co. (Shanghai, China) following their standard IHC service protocol. Stained cores were digitized, and VGLL4 protein expression was quantified using the APTime (v3.0) image analysis system as the mean optical density (MOD) of DAB staining within each tissue core. This quantitative, software-based approach was used to minimize subjective scoring bias.

### 2.3. Cell Lines and Culture Conditions

The human ccRCC cell lines (Caki-1, OS-RC-2, and ACHN) and the normal renal tubular epithelial cell line (HK-2) were obtained from the Cell Bank of the Chinese Academy of Sciences. All cell lines were maintained at 37 °C in a 5% CO_2_ atmosphere in their recommended medium containing 10% fetal bovine serum and 1% penicillin/streptomycin: Caki-1 in McCoy’s 5A, OS-RC-2 in RPMI-1640, and both ACHN and HK-2 in DMEM. For the rescue experiments, cells were treated with GA-017 (3 μM; TargetMol, Boston, MA, USA) or CHIR-99021 (3 μM; TargetMol) to activate the Hippo/YAP or Wnt/β-catenin signaling cascades, respectively.

### 2.4. Plasmid and siRNA Transfection

The overexpression plasmid and small interfering RNAs (siRNAs) targeting VGLL4 were designed and synthesized by GenePharma Co., Ltd. (Shanghai, China). The VGLL4 overexpression plasmid was constructed using the pEX-3 vector (pGCMV/MCS/Neo). The sequences of the VGLL4-specific siRNAs were as follows: VGLL4-Homo-318 GCAAGAGGAAGUUCAGCAUTT;

VGLL4-Homo-220 AAACGCGAAGCUGCUCUCATT;

VGLL4-Homo-802 AGGAGCCUGGGCAAGAAUUTT;

VGLL4-Homo-899 ACACGUGGCUCCAGAUCAATT.

For transfection, cells were seeded overnight and grown to approximately 70% confluency. According to the manufacturer’s instructions, plasmids or siRNA were transfected into cells using Lipofectamine 2000 reagent (Invitrogen, Waltham, MA, USA) and cultured until harvest for each experiment.

### 2.5. RNA Isolation and Quantitative Real-Time PCR (qRT-PCR)

Total RNA was extracted from cells using the RNA Easy Fast Tissue/Cell Kit (Tiangen, Beijing, China). Complementary DNA (cDNA) was synthesized from the extracted RNA using the PrimeScript^TM^ FAST RT Kit (with gDNA Eraser) (Takara, Kusatsu, Japan). Quantitative PCR was performed to measure mRNA expression levels using the TB Green^®^ Premix Ex Taq^TM^ II FAST qPCR Kit (Takara, Japan) on a LightCycler^®^ 480 Detection System (Roche, Diagnostics, Indianapolis, IN, USA). The relative expression of VGLL4 was normalized to GAPDH and calculated using the 2^−ΔΔCT^ method. All primers for VGLL4 and GAPDH were synthesized by Sangon Biotech (Shanghai, China). VGLL4: forward primer, 5′-ACCGTCTACCAGTAAGCCCTTG-3′ and reverse primer, 5′-GCTCCTGGCTCTTCAACTTCAC-3′; GAPDH: forward primer, 5′-CCAGCAAGAGCACAAGAGGAAG-3′ and reverse primer, 5′-GTCTACATGGCAACTGTGAGGAG-3′.

### 2.6. Western Blot and Antibodies

Cells were lysed on ice using RIPA lysis buffer (Beyotime, Shanghai, China) supplemented with PMSF and phosphatase inhibitors, and the supernatant was collected by centrifugation. Protein concentration was determined using a BCA protein assay kit (Beyotime, Shanghai, China). Subsequently, the lysates were denatured by boiling at 100 °C for 10 min in 5× SDS loading buffer. The denatured proteins were separated by 10% SDS-PAGE and then transferred onto a PVDF membrane (Millipore, Billerica, MA, USA). The membrane was blocked with 5% BSA for 2 h at room temperature, followed by incubation with primary antibodies at 4 °C overnight. After washing, the membrane was incubated with secondary antibodies for 1 h at room temperature. Specific protein signals were visualized using an enhanced chemiluminescence (ECL) substrate (Biosharp, Beijing, China) on a ChemiScope 6000 chemiluminescence imaging system (CLiNX, Shanghai, China). The following primary antibodies were used: VGLL4 (A18248, ABclonal, 1:1000), β-Tubulin (TA-10, ZSGB-BIO, Beijing, China, 1:1000), GAPDH (EM1101, HUABIO, Hangzhou, China, 1:20,000); phospho-YAP(S127) (ABP50490, Abbkine, Atlanta, GA, USA, 1:1000), Survivin (AB76424, Abcam, Cambridge, UK, 1:5000), CTGF (23936-1-AP, Proteintech, Rosemont, IL, USA, 1:1000), β-catenin (ab246504, Abcam, 1:1000), phospho-β-catenin (S37) (ab75777, Abcam, 1:1000), Cyclin D1 (ab134175, Abcam, 1:10,000), GSK3β (ab32391, Abcam, 1:5000), E-cadherin (60335-1-lg, Proteintech, 1:4000), N-cadherin (66219-1-lg, Proteintech, 1:20,000), and MMP2 (66366-1-lg, Proteintech, 1:3000). HRP-conjugated goat anti-rabbit or anti-mouse IgG antibodies were used as secondary antibodies.

### 2.7. Cell Viability Assay (MTT Assay)

ccRCC cells in the logarithmic growth phase were seeded into 96-well plates and transfected the following day. At each designated time point, 20 µL of MTT reagent (5 mg/mL) was added to each well, followed by incubation at 37 °C for 4 h. Thereafter, 50 µL of acidified SDS solution (20%, *w*/*v*) was added to each well and the plates were incubated overnight. The absorbance was measured at a wavelength of 570 nm using a SpectraMax iD5 multi-mode microplate reader (Molecular Devices, San Jose, CA, USA). Data analysis was performed using GraphPad Prism software (version 9.0).

### 2.8. Colony Formation Assay

Following transfection with either the VGLL4-overexpressing plasmid or VGLL4-targeting siRNA, ccRCC cells were seeded into 6-well plates at a density of 2000 cells per well. The culture medium was replaced every three days. After 10 days of culture, the cells were fixed with 4% paraformaldehyde for 30 min and then stained with crystal violet staining solution (Beyotime, Shanghai, China) for 15 min. The stained colonies were air-dried at room temperature and subsequently imaged.

### 2.9. Cell Proliferation Assay (EdU Staining)

Transfected ccRCC cells were seeded into 96-well plates at a density of 2000 cells per well. After 24 h, cell proliferation was assessed using the BeyoClick^TM^ EdU-555 Cell Proliferation Detection Kit (Beyotime, Shanghai, China) according to the manufacturer’s instructions. Images were acquired using a fluorescence microscope (Olympus, Tokyo, Japan) for visualization and analysis.

### 2.10. Cell Cycle and Apoptosis Assays

For cell cycle analysis, transfected ccRCC cells were harvested, washed twice with ice-cold PBS, and fixed in 70% ethanol at 4 °C overnight. Subsequently, the ethanol was removed via centrifugation, and the cells were resuspended in PI/RNase Staining Buffer (BD Biosciences, San Jose, CA, USA). After incubation for 30 min at room temperature in the dark, the cell cycle distribution was assessed using a flow cytometer (Agilent, Santa Clara, CA, USA). Cell apoptosis was analyzed with an Annexin V-FITC/PI Apoptosis Detection Kit (KeyGEN BioTECH, Nanjing, China) in accordance with the manufacturer’s protocol.

### 2.11. Wound Healing Assay

Transfected ccRCC cells were plated in 6-well plates and cultured until they reached full confluence. A straight scratch was introduced into the cell monolayer using a sterile 200 µL pipette tip. After gently washing with PBS to remove dislodged cells, serum-free medium was added to each well. Images of the wound area were captured at 0 and 24 h using an inverted microscope (Olympus, Tokyo, Japan). The migration rate was quantitatively evaluated by measuring the wound closure with ImageJ software 1.54r.

### 2.12. Cell Invasion Assay (Transwell)

For the invasion assay, Matrigel (Corning, Corning, NY, USA) was diluted in serum-free medium (1:15) and 60 µL was coated onto the Transwell membrane. After polymerization at 37 °C for 2–3 h, transfected ccRCC cells were harvested, counted, and resuspended in serum-free medium. Then, 6000 cells in suspension were added to the upper chamber. The lower chamber was filled with medium containing 10% FBS as a chemoattractant. After 24 h of incubation, non-invaded cells on the upper membrane surface were removed with a cotton swab. Invaded cells on the lower surface were fixed with 4% paraformaldehyde (15 min), stained with crystal violet (10 min), washed with PBS, and air-dried. Images were captured using an inverted microscope. The migration assay was performed identically, but without Matrigel coating.

### 2.13. Establishment of Stable VGLL4-Overexpressing Cell Line

The stable VGLL4-overexpressing cell line was generated by GenePharma Co., Ltd. (Shanghai, China) using a lentivirus-mediated delivery system. Briefly, the full-length coding sequence of human VGLL4 was cloned into the lentiviral overexpression vector LV5-EF1a-GFP-Puro, which expresses the gene under the control of the EF1α promoter and contains GFP and puromycin resistance markers. The recombinant lentivirus was packaged and subsequently used to infect target cells. Stable infectants were selected by culturing the transduced cells under puromycin selection pressure (1.5 μg/mL). Infection efficiency was monitored via GFP fluorescence, and puromycin-resistant colonies were expanded for further validation of VGLL4 overexpression using qRT-PCR and Western blot analysis.

### 2.14. Real-Time Cell Analysis (RTCA)

Real-time, label-free monitoring of cell viability was conducted using the xCELLigence system (Agilent, Santa Clara, CA, USA). Initially, 50 µL of culture medium was added to each well of a 16-well E-Plate to acquire a baseline impedance measurement. Then, 4000 cells were seeded per well and cultured at 37 °C with 5% CO_2_. After cell attachment, the system continuously recorded cellular status. The results are expressed as the delta cell index, representing normalized changes in electrical impedance that reflect variations in cell number, morphology, and adhesion over time.

### 2.15. Immunofluorescence

Immunofluorescence was performed using the BeyoTSA^TM^ Dual-color Multiple Fluorescence Staining Kit (488/594/DAPI, Beyotime, Shanghai, China) according to the manufacturer’s protocol. Briefly, cells were fixed, permeabilized, and blocked, then incubated sequentially with primary antibodies against phospho-YAP (ABP50490, Abbkine, 1:300) and VGLL4 (A18248, Abclonal, 1:300), followed by HRP-conjugated secondary antibodies. Tyramide signal amplification was performed sequentially using Tyramide-594 (for phospho-YAP, red signal) and Tyramide-488 (for VGLL4, green signal), with an antibody stripping step between each round. Nuclei were stained with DAPI. Images were acquired using a fluorescence microscope (Olympus, Tokyo, Japan).

### 2.16. RNA Sequencing and Bioinformatic Analysis

Total RNA was isolated from VGLL4-overexpressing and wild-type Caki-1 cells using TRIzol reagent, with three independent biological replicates per group. RNA library preparation and transcriptome sequencing were performed by OE Biotech Co., Ltd. (Shanghai, China). Differentially expressed genes (DEGs) were identified using the DESeq2 package in R, with cutoff criteria set as |log_2_FoldChange| > 1 and q-value (Benjamini–Hochberg FDR-adjusted *p* value) < 0.05. Subsequent bioinformatics analysis of DEGs was conducted using the OECloud platform.

### 2.17. In Vivo Xenograft Tumor Model

All animal experiments were reviewed and approved by the Institutional Animal Care and Use Committee of Sichuan Provincial People’s Hospital (Approval No.: 202592, Approval Date: 13 February 2025). Six-week-old male NOD/SCID mice (Vital River Laboratory, Beijing, China) were acclimated for one week under SPF conditions. Mice were randomly divided into the Caki-1-VGLL4 and Caki-1-Vector groups using a computer-generated randomization sequence (*n* = 6 per group), and group allocation was concealed until the start of cell inoculation. Each mouse received a subcutaneous injection of 5 × 10^6^ cells/100 μL into the right flank. Tumor growth was monitored from one week post-injection by measuring tumor dimensions every three days with a caliper. Tumor volume was calculated as: volume (mm^3^) = (length × width^2^) × 0.52. The experiment was terminated when the mean tumor volume in either group reached 1500 mm^3^; mice were then euthanized and tumors were collected for subsequent analysis.

### 2.18. Immunohistochemical (IHC) Staining

Tumor tissues were fixed in 10% neutral buffered formalin and paraffin-embedded. Sections were prepared following standard histological procedures, including deparaffinization, antigen retrieval, and immunostaining. The following primary antibodies were applied: VGLL4 (A18248, ABclonal; 1:100), CD31 (SU03-59, HUABIO; 1:500), and Ki67 (ST50-01, HUABIO; 1:200). Hematoxylin (Beyotime, Shanghai, China) was used for nuclear counterstaining. Whole-slide images were captured using a KF-PRO-005-EX digital slide scanner (KFBIO, Ningbo, China).

### 2.19. Statistical Analysis

All experimental data were analyzed with GraphPad Prism (v9.0). Results are presented as the mean ± standard deviation (SD), with each experiment repeated at least three times. Statistical comparisons between two groups or among multiple groups were performed using unpaired *t*-tests or one-way ANOVA, respectively. Data from the TCGA database were evaluated using R (version 4.2.2), except for automatic data analysis on websites. A *p*-value of less than 0.05 was considered statistically significant.

## 3. Results

### 3.1. VGLL4 Expression and Clinicopathologic Correlations in ccRCC

**Figure 1:**
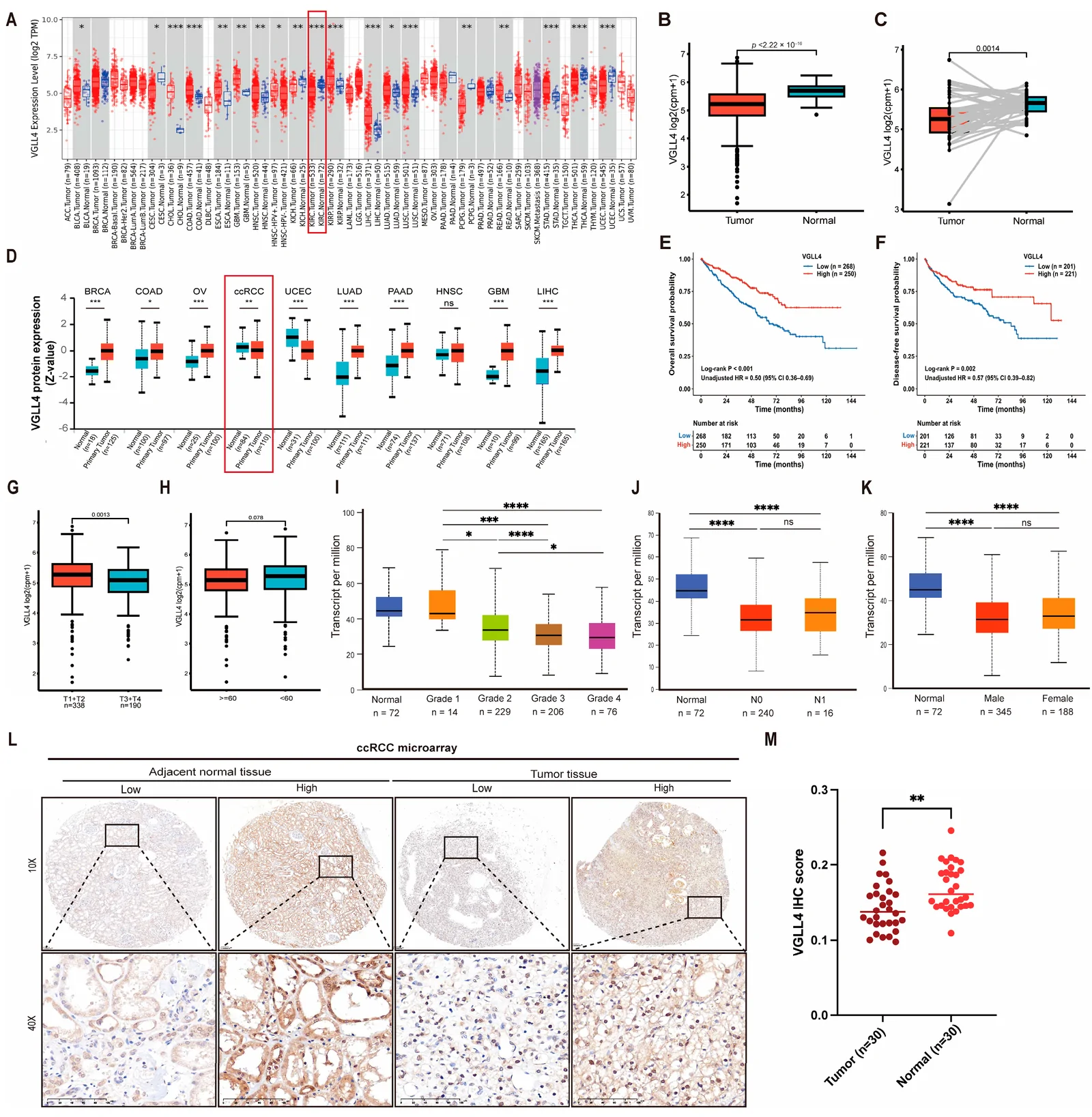
VGLL4 is downregulated in ccRCC and correlates with a favorable patient prognosis. (**A**) VGLL4 expression in pan-cancer analysis (TIMER database). (**B**) VGLL4 expression in ccRCC and normal kidney tissues from the TCGA database. (**C**) Comparison of VGLL4 expression between 72 paired ccRCC tumors and adjacent normal tissues in the TCGA cohort. (**D**) Protein expression levels of VGLL4 across various cancer types from the UALCAN database. (**E**,**F**) Kaplan–Meier survival analysis showing the association between VGLL4 expression and overall survival (**E**) or disease-free survival (**F**) in ccRCC patients from the TCGA-KIRC cohort, with stratification by VGLL4 expression using a cutoff value of 15.9 TPM determined with X-tile 3.6.1. (**G**,**H**) Correlation of VGLL4 expression with T stage (**G**) and age (**H**) in the TCGA ccRCC cohort, analyzed using R software. (**I**–**K**) Correlation of VGLL4 expression with tumor grade (**I**), nodal metastasis status (**J**), and patient gender (**K**), analyzed using the UALCAN database. (**L**) Representative images of VGLL4 IHC staining from a human tissue microarray. (**M**) Statistical analysis of VGLL4 IHC staining in ccRCC and matched normal tissues from the tissue microarray (*n* = 30). Unpaired *t*-tests were used to compare the difference between two groups. Data are shown as the mean ± SD. * *p* < 0.05, ** *p* < 0.01, *** *p* < 0.001, **** *p* < 0.0001 indicate significant differences, and *p* > 0.05 means no significance difference (ns).

To investigate the expression profile of VGLL4 and its potential link to ccRCC progression, we initially conducted a pan-cancer analysis of VGLL4 mRNA expression using the TIMER2 database. VGLL4 mRNA levels were lower in most tumor tissues compared to their corresponding normal counterparts, including ccRCC (Figure 1A,B).

Paired-sample analysis further confirmed a significant reduction in VGLL4 in ccRCC tumors compared with matched adjacent normal tissues from the same patients (Figure 1C). Consistent with these findings, protein-level data from UALCAN and the Human Protein Atlas also indicated marked downregulation of VGLL4 in ccRCC (Figure 1D and Figure S1). Univariate survival analysis revealed that ccRCC patients with higher VGLL4 expression experienced a favorable prognosis, characterized by longer overall survival (OS; Log-rank *p* < 0.001, unadjusted HR = 0.50, 95% CI: 0.36–0.69) and disease-free survival (DFS; Log-rank *p* = 0.002, unadjusted HR = 0.57, 95% CI: 0.39–0.82) (Figure 1E,F). The optimal cutoff for VGLL4 expression (TPM, primary tumor) was determined by X-tile 3.6.1 and set at 15.9 (Table S1), which was used to dichotomize patients into VGLL4-high and VGLL4-low groups. In multivariate analysis adjusting for age, sex, pathologic stage, and histologic grade, high VGLL4 expression remained a predictor of improved OS (HR = 0.63, 95% CI: 0.45–0.87, *p* = 0.006; Table S2). After multivariate adjustment, the association with DFS was not statistically significant (HR = 0.72, 95% CI: 0.49–1.05, *p* = 0.089; Table S3), although a trend toward reduced risk was observed. We next examined the correlation between VGLL4 expression and clinicopathological parameters of ccRCC patients. Analysis of the TCGA dataset showed that VGLL4 expression was modestly downregulated in patients with higher T-stage and in those aged ≥60 years (Figure 1G,H). Extended analysis using the UALCAN database further demonstrated significantly lower VGLL4 expression in patients with higher tumor grade, whereas no statistically significant associations were observed with nodal metastasis status or gender (Figure 1I–K). To independently validate these findings, we performed immunohistochemical staining on a tissue microarray containing 30 paired ccRCC and adjacent normal specimens. Representative staining images are shown in Figure 1L and quantitative analysis confirmed a statistically significant reduction in VGLL4 protein in tumor tissues compared with matched normal controls (*p* < 0.01, Figure 1M). Taken together, these results indicate that VGLL4 is substantially downregulated in ccRCC at both mRNA and protein levels, and its reduced expression correlates with adverse clinicopathological features and poorer overall survival, supporting its potential role as a negative prognostic-associated biomarker in ccRCC.

### 3.2. VGLL4 Suppresses Cell Viability and Clone Formation of ccRCC Cells

**Figure 2:**
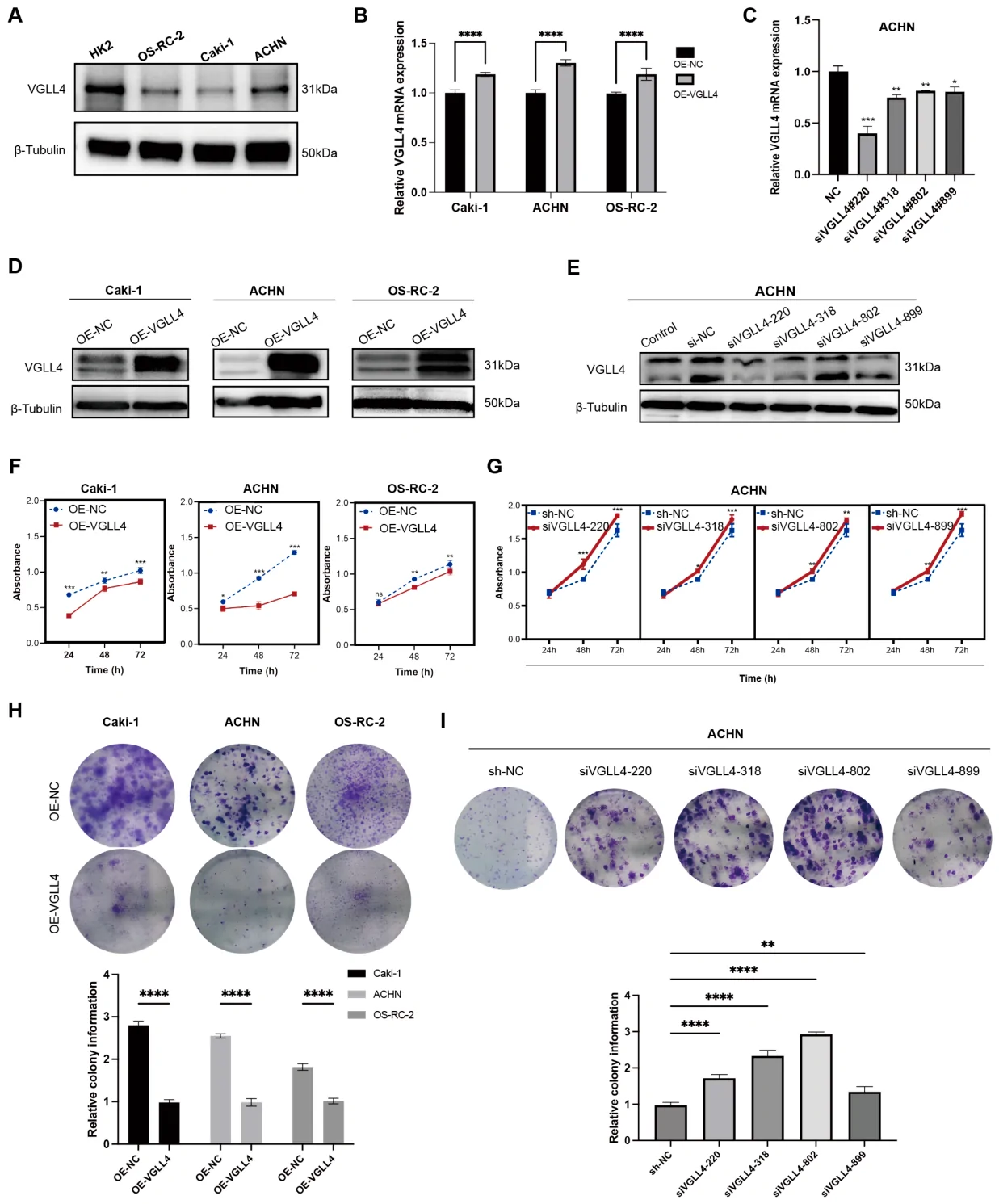
VGLL4 overexpression suppresses cell viability and colony formation of ccRCC cells. (**A**) VGLL4 protein levels in a normal renal cell line (HK-2) and three ccRCC cell lines (Caki-1, ACHN, and OS-RC-2) were determined by Western blotting. (**B**–**E**) Validation of VGLL4 overexpression and knockdown efficiency in ccRCC cells by RT-qPCR (*n* = 8) and Western blot. (**F**,**G**) The effect of VGLL4 overexpression and knockdown on cell viability was assessed by MTT assay at different time points (*n* = 6). (**H**,**I**) The proliferation capacity of ccRCC cells with VGLL4 overexpression or knockdown was evaluated through colony formation assays (*n* = 3). Data are shown as the mean ± SD. * *p* < 0.05, ** *p* < 0.01, *** *p* < 0.001, **** *p* < 0.0001 indicate significant difference, and *p* > 0.05 means no significance difference (ns).

To investigate the role of VGLL4 in ccRCC progression, we performed cell viability assays on ccRCC cells with VGLL4 overexpression or knockdown. Among the cell lines used, the ccRCC cells (Caki-1, ACHN, and OS-RC-2) exhibited lower VGLL4 expression compared to the normal renal tubular epithelial cell line HK-2, which is consistent with the results aforementioned (Figure 2A).

As shown in Figure 2B,D, transfection with a VGLL4-overexpression plasmid substantially increased its mRNA and protein levels in all three ccRCC cell lines. In contrast, VGLL4 siRNAs markedly reduced VGLL4 expression at both the mRNA and protein levels in ACHN cells (Figure 2C,E). Functionally, VGLL4 overexpression significantly suppressed cell viability in Caki-1, ACHN, and OS-RC-2 cells at 48 and 72 h (Figure 2F). We further selected ACHN cells for loss-of-function validation because of its relatively high VGLL4 expression and reproducible knockdown efficiency. Conversely, VGLL4 knockdown moderately enhanced the viability of ACHN cells (Figure 2G). The same results could also be observed in colony formation assays, in which VGLL4 overexpression notably impaired the clonogenic ability of ccRCC cells (Figure 2H), whereas its knockdown promoted colony formation (Figure 2I). Taken together, these results demonstrate that VGLL4 acts as a potent suppressor of ccRCC cell proliferation and clonogenicity, supporting its tumor-suppressive function in this malignancy.

### 3.3. VGLL4 Overexpression Inhibits Proliferation and Induces Cell Cycle Arrest of ccRCC Cells

**Figure 3:**
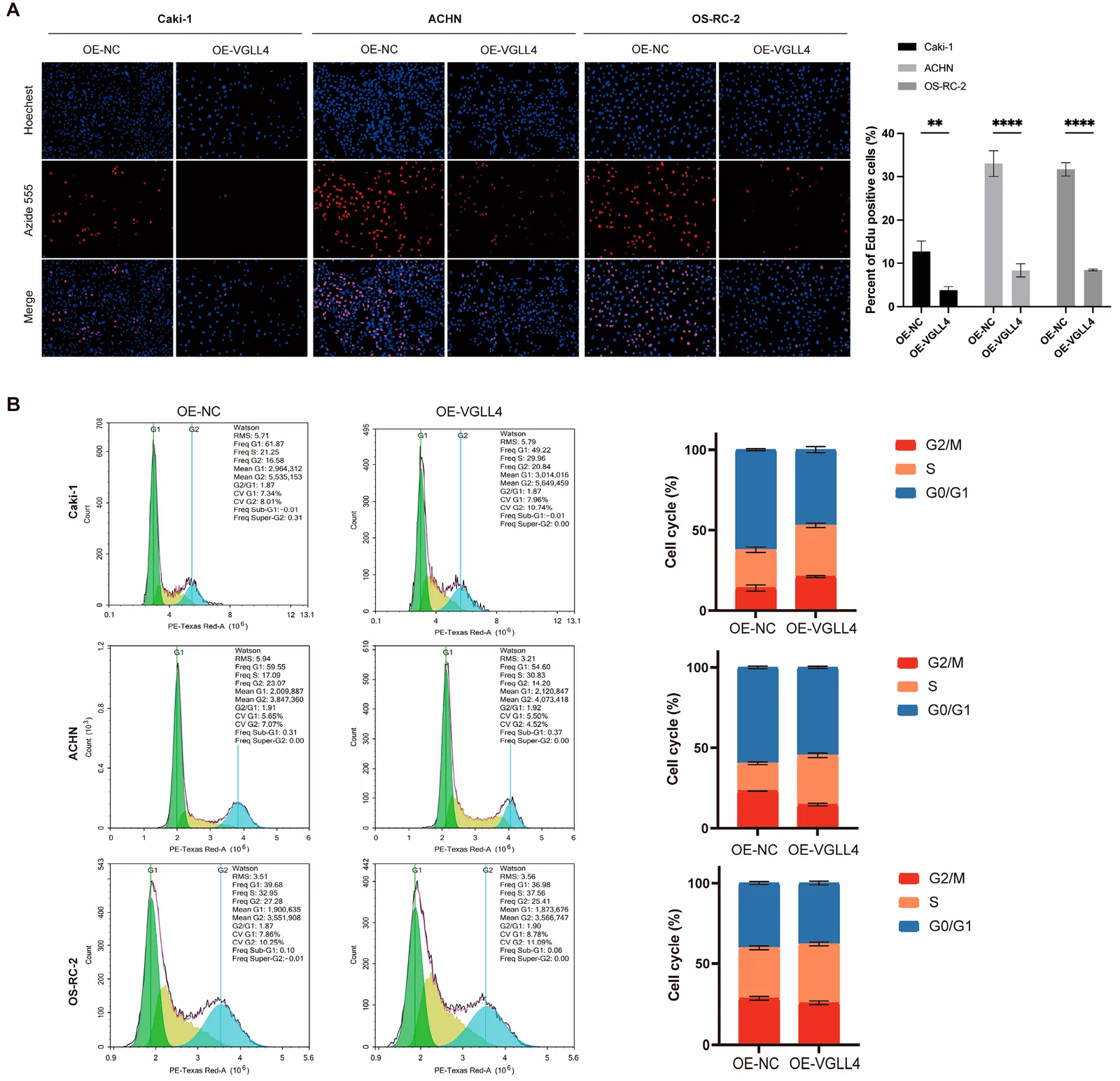
Upregulation of VGLL4 inhibited proliferation and induced cell cycle arrest in ccRCC cells. (**A**) EdU incorporation assay of ccRCC cells transfected with control or VGLL4 plasmid. Nuclei were counterstained with Hoechst 33342 (blue), and EdU-positive proliferating cells are shown in red (*n* = 3). (**B**) Flow cytometry analysis of cell cycle distribution (*n* = 3). Data are shown as the mean ± SD. ** *p* < 0.01 and **** *p* < 0.0001 indicate significant difference.

To further validate the inhibitory effect of VGLL4 on ccRCC cell proliferation, we employed EdU incorporation assay and flow cytometry to analyze DNA replication activity and cell cycle distribution in Caki-1, ACHN, and OS-RC-2 cells overexpressing VGLL4. In the EdU assay, VGLL4 overexpression significantly reduced the percentage of EdU-positive (red) cells (Figure 3A), confirming its potent anti-proliferative effect.

Flow-cytometric analysis further showed that VGLL4 upregulation led to a discernible accumulation of cells in S phase (Figure 3B), suggesting that VGLL4 induces a partial S-phase arrest. Notably, apoptosis was also examined by Annexin V/PI double staining; however, there was no significant difference in the apoptosis rate between the vehicle and VGLL4-overexpressing groups. Taken together, these results indicate that VGLL4 inhibits ccRCC cell growth mainly through suppressing cellular proliferation activity rather than inducing apoptosis.

### 3.4. VGLL4 Overexpression Inhibits the Migration and Invasion of ccRCC Cells

**Figure 4:**
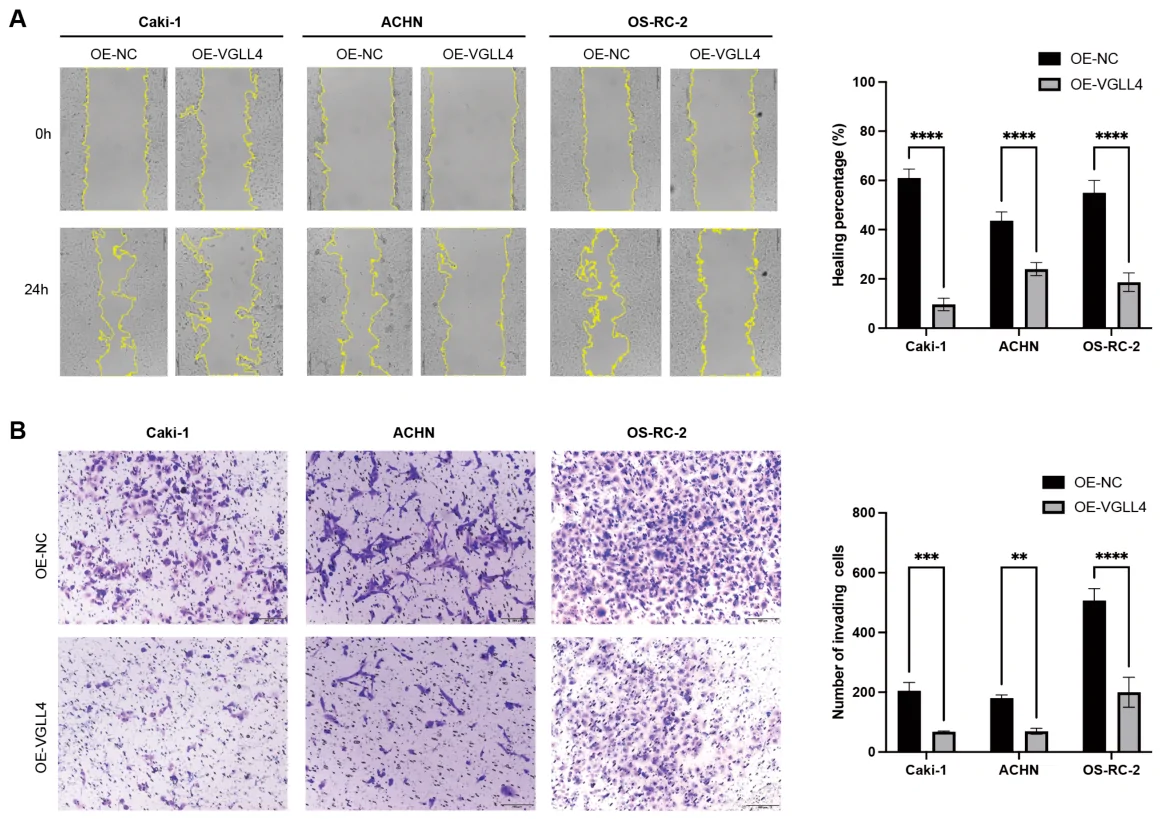
VGLL4 restrained the migration and invasion of ccRCC cells in vitro. (**A**) Representative images of wound healing assays with quantification of the healing percentage (*n* = 3). (**B**) Representative images of Transwell invasion assays with quantification of invaded cells (*n* = 3). Scale bars represent 100 µm. Data are shown as the mean ± SD. ** *p* < 0.01, *** *p* < 0.001, and **** *p* < 0.0001 indicate significant difference.

To assess the impact of VGLL4 on ccRCC cell motility and invasion, wound-healing and Transwell invasion assays were conducted. VGLL4 overexpression significantly impeded cell migration, as evidenced by reduced wound closure compared to the control group (Figure 4A). Similarly, in the Transwell invasion assay, upregulation of VGLL4 markedly reduced the invasive capability of ccRCC cells (Figure 4B). These results indicate that VGLL4 acts as a suppressor of migratory and invasive phenotypes in ccRCC, suggesting its potential role in restraining tumor metastasis.

### 3.5. Establishment and Phenotypic Validation of VGLL4 Stable Expression Cell Line

**Figure 5:**
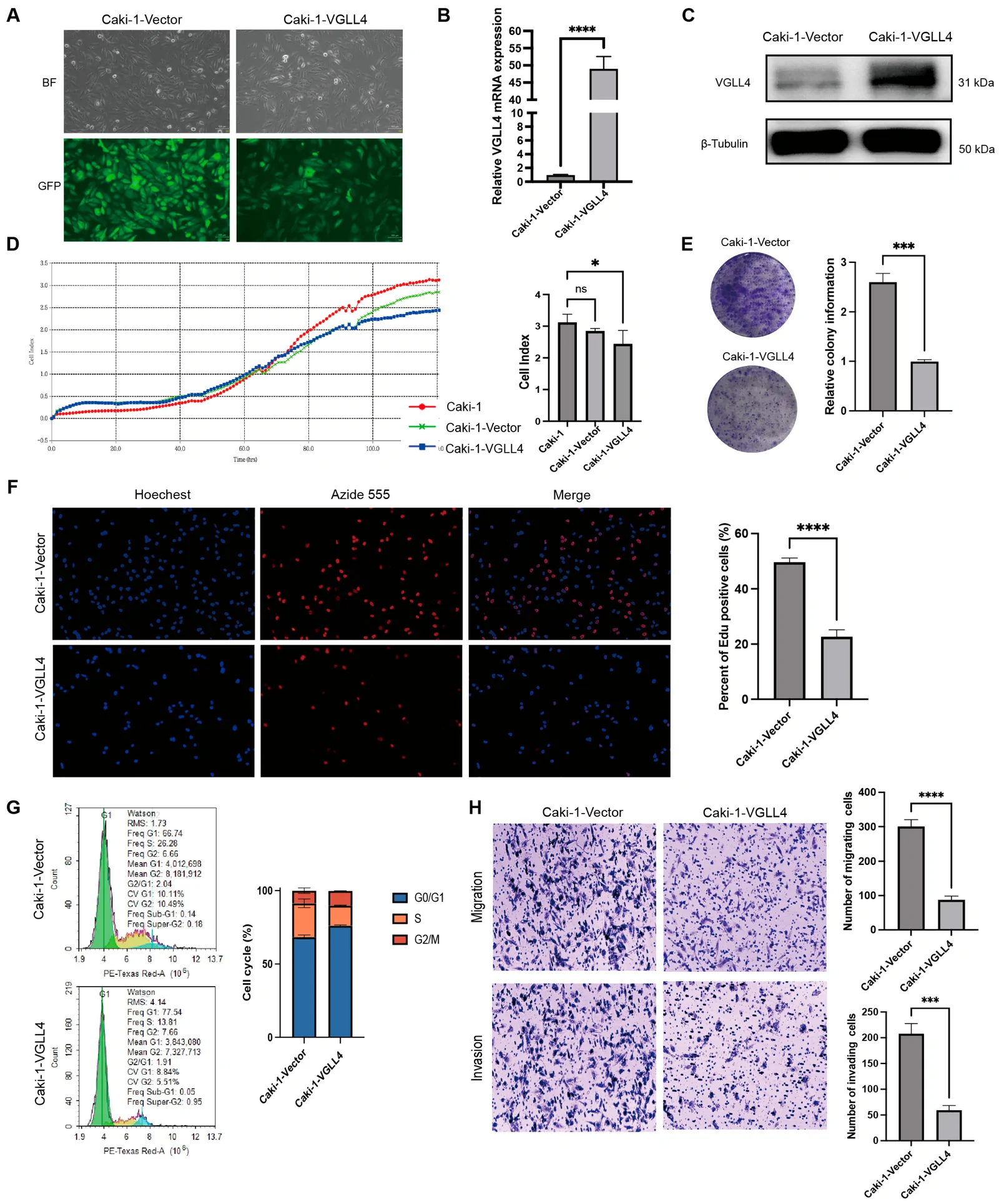
Establishment and phenotypic validation of VGLL4 stable expression cell line. (**A**) Fluorescence microscopy images showing lentiviral transduction efficiency in Caki-1 cells. (**B**,**C**) RT-qPCR (*n* = 8) and Western blot analysis confirming VGLL4 expression in stably infected Caki-1 cells. (**D**–**F**) RTCA (*n* = 4), colony formation (*n* = 3) and EdU assays (*n* = 3) assessing the effects of VGLL4 overexpression on the proliferative capacity of Caki-1 cells. (**G**) Flow cytometric analysis of cell cycle distribution in stably transduced Caki-1 cells (*n* = 3). (**H**) Transwell migration and invasion assays assessing the migratory and invasive capacity of stably transduced Caki-1 cells (*n* = 3). Data are shown as the mean ± SD. * *p* < 0.05, *** *p* < 0.001, **** *p* < 0.0001 indicate significant difference, and *p* > 0.05 means no significance difference (ns).

To generate a stable cellular model for subsequent in vivo studies and to consolidate the phenotypic effects observed upon transient VGLL4 overexpression, we selected the Caki-1 cell line, which exhibits relatively low endogenous VGLL4 expression (Figure 2A), for model establishment. As depicted in Figure 5A–C, we successfully constructed the Caki-1 cell model stably overexpressing VGLL4 (Caki-1-VGLL4) via lentiviral transfection.

Caki-1-VGLL4 cells displayed a delayed cell growth rate compared with the parental cells and vector control cells (Caki-1-Vector) in the RTCA analysis (Figure 5D). Consistent with the results of transient transfection, colony formation and EdU incorporation assays also demonstrated decreased clonogenic ability (Figure 5E) and proliferative activity of Caki-1-VGLL4 cells (Figure 5F). Flow cytometric analysis revealed that stable VGLL4 overexpression induced cell cycle arrest at the G0/G1 phase (Figure 5G), which differed from the S-phase arrest observed in the previous transient overexpression cells (Figure 3B). The discrepancy likely arises from differences in expression magnitude, time window, and cellular adaptive status between the two systems: acute high-level transient expression triggers stress-associated S-phase arrest, whereas sustained lower-level lentiviral expression after long-term selection favors classical G0/G1 arrest and may enrich for adapted subpopulations. Additionally, the migratory and invasive capabilities of Caki-1 cells were also significantly restrained following stable VGLL4 transfection (Figure 5H). These results further validate the tumor-suppressive function of VGLL4 in ccRCC.

### 3.6. VGLL4 Constrains ccRCC Progression Through Coregulating the Hippo/YAP and Wnt/β-Catenin Signaling Pathways

**Figure 6:**
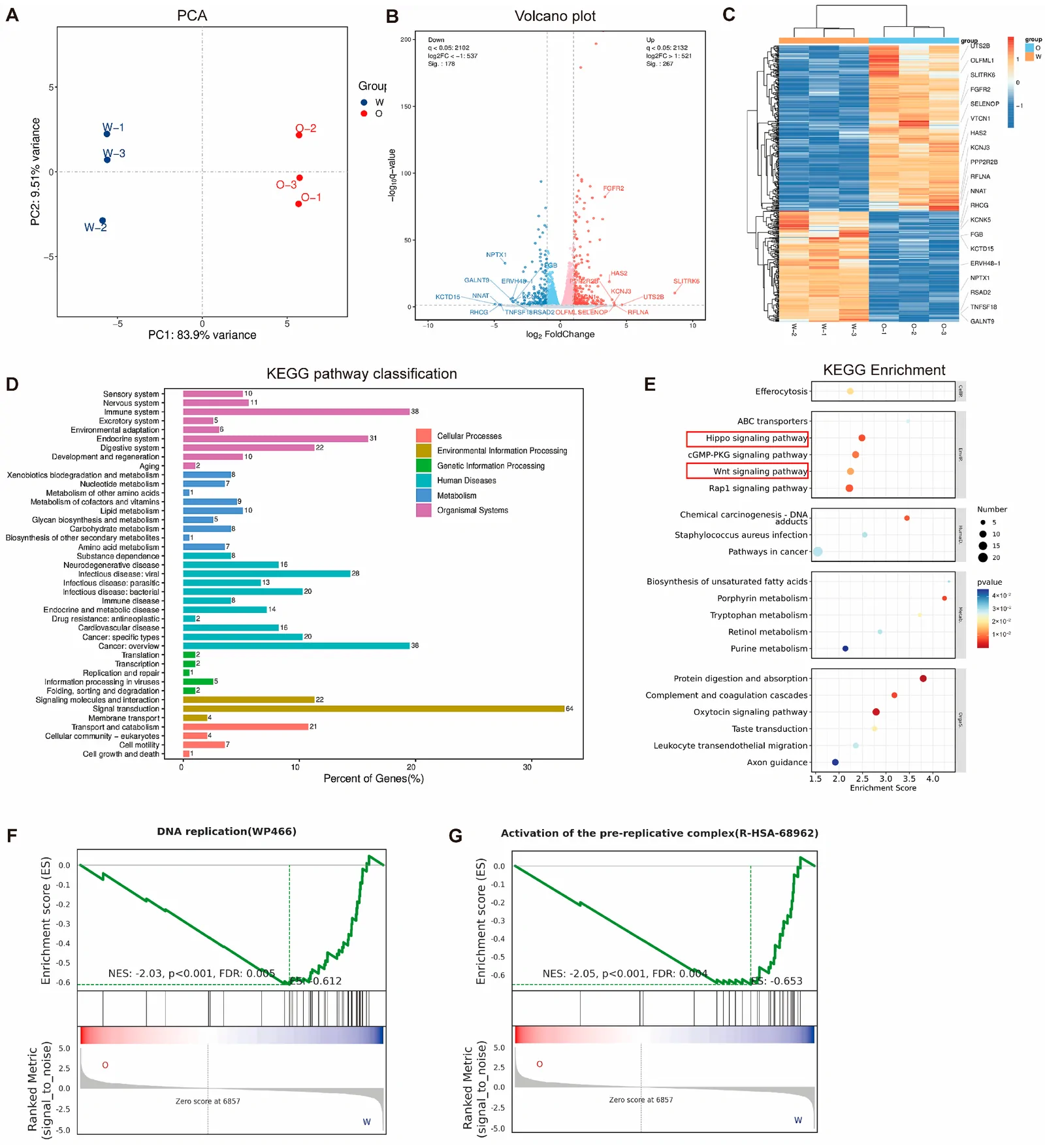
Transcriptomic Profiling Reveals VGLL4-Regulated Genes and Pathways in ccRCC. (**A**) Principal component analysis of RNA sequencing data demonstrates distinct transcriptomic profiles between the two groups. (**B**,**C**) The volcano plot (**B**) and heatmap (**C**) display the DEGs between parental and VGLL4-overexpressing Caki-1 cells. (**D**) KEGG pathway classification analysis of DEGs. (**E**) KEGG pathway enrichment analysis (top 20) of DEGs. (**F**,**G**) The GSEA results show suppression of DNA replication (**F**) and activation of the pre-replicative complex (**G**) upon VGLL4 overexpression.

To investigate the potential molecular mechanisms by which VGLL4 suppresses ccRCC, we performed RNA transcriptome sequencing on Caki-1-VGLL4 and wild-type Caki-1 cells. As displayed in Figure 6A, a significant difference could be observed between the Caki-1-VGLL4 and parental Caki-1 cells according to the principal component analysis (PCA).

A total of 445 differentially expressed genes (DEGs) were identified, comprising 267 upregulated genes and 178 downregulated genes (Figure 6B,C). Kyoto Encyclopedia of Genes and Genomes (KEGG) pathway classification analysis revealed that DEGs were primarily enriched in the pathways related to cancer, signal transduction, and immune system (Figure 6D). In the top 20 KEGG enrichment pathways, Hippo and Wnt signaling cascades are the crucial pathways involved in tumor cell signal transduction (Figure 6E) [16,17,18,19]. Notably, gene set enrichment analysis (GSEA) revealed significant downregulation of hallmark pathways related to DNA replication and pre-replicative complex activation in Caki-1-VGLL4 cells (Figure 6F,G and Figure S2A,B), aligning with the observed suppression of proliferation and cell-cycle arrest.

**Figure 7:**
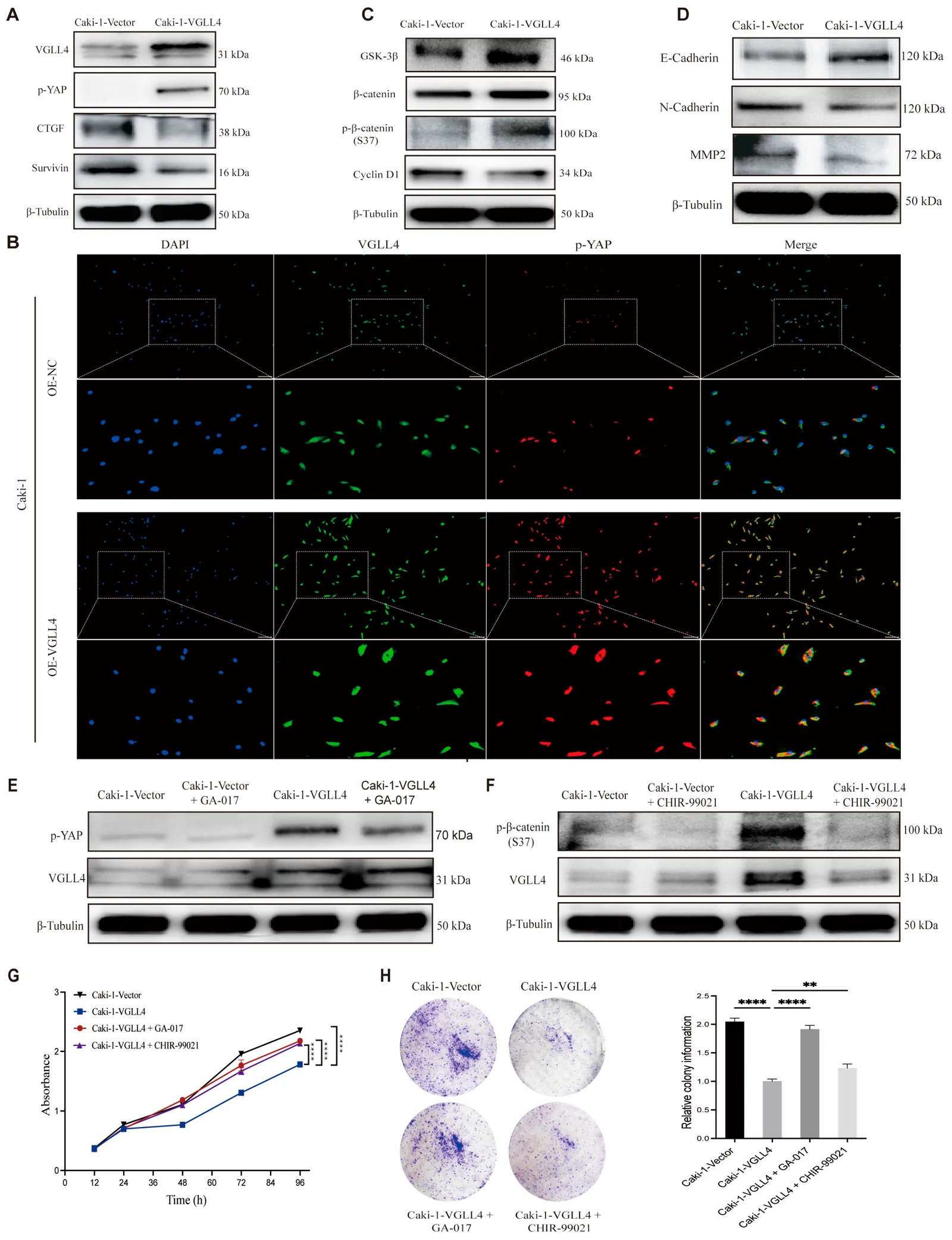
VGLL4 suppresses ccRCC progression through dual modulation of the Hippo/YAP and Wnt/β-catenin pathways. (**A**) Western blot analysis of Hippo pathway components. (**B**) Immunofluorescence staining for phosphorylated YAP (p-YAP, red) and VGLL4 (green) in Caki-1 cells. Nuclei are stained with DAPI (blue) (scale bar represents 100 μm). (**C**) Western blot analysis of Wnt/β-catenin pathway components. (**D**) Western blot analysis of epithelial–mesenchymal transition (EMT) markers. (**E**,**F**) Western blot analysis of rescue experiments. Treatment with the YAP activator GA-017 (**E**) or the β-catenin activator CHIR-99021 (**F**) reverses the increase in p-YAP or p-β-catenin levels induced by VGLL4 overexpression in Caki-1 cells. (**G**,**H**) The impaired proliferative and clonogenic capacities of Caki-1-VGLL4 cells are partially rescued by treatment with the GA-017 or CHIR-99021, as assessed by MTT (*n* = 6) and colony formation assays (*n* = 3). Data are shown as the mean ± SD. ** *p* < 0.01 and **** *p* < 0.0001 indicate significant difference.

Based on the transcriptomic profiling suggesting the involvement of the Hippo and Wnt signaling pathways in VGLL4-mediated anti-ccRCC effects, we performed validation using Western blot analysis. As shown in Figure 7A, Caki-1-VGLL4 cells showed elevated expression of YAP phosphorylation compared with the vector cells, suggesting that VGLL4 may trigger a positive feedback loop that inactivates YAP. This inactivation, combined with VGLL4’s transcriptional antagonism of YAP, effectively suppressed the expression of key downstream target proteins of YAP, including Survivin and CTGF (Figure 7A).

Consistently, immunofluorescence analysis visually demonstrated a pronounced and widespread enhancement of phosphorylated YAP immunostaining specifically in VGLL4-overexpressing cells compared to control cells, further supporting the role of VGLL4 in inducing YAP phosphorylation and subsequent inactivation (Figure 7B). Regarding the Wnt/β-catenin pathway, Caki-1-VGLL4 cells displayed increased levels of GSK-3β, and β-catenin phosphorylation, indicating that VGLL4 promotes β-catenin degradation while suppressing active β-catenin. Consistently, this inhibition significantly reduced the expression of its downstream protein Cyclin D1, a critical regulator of cell cycle and cell proliferation (Figure 7C). Given the established association between these pathways and epithelial–mesenchymal transition (EMT) [20,21,22], along with the observed inhibitory effects of VGLL4 on cell migration and invasion, we further examined EMT-related markers by Western blot analysis. As depicted in Figure 7D, VGLL4 overexpression significantly upregulated the epithelial marker E-cadherin and downregulated the mesenchymal markers N-cadherin, as well as the matrix metalloproteinase MMP2, supporting an inhibitory role for VGLL4 in the EMT process.

**Figure 8:**
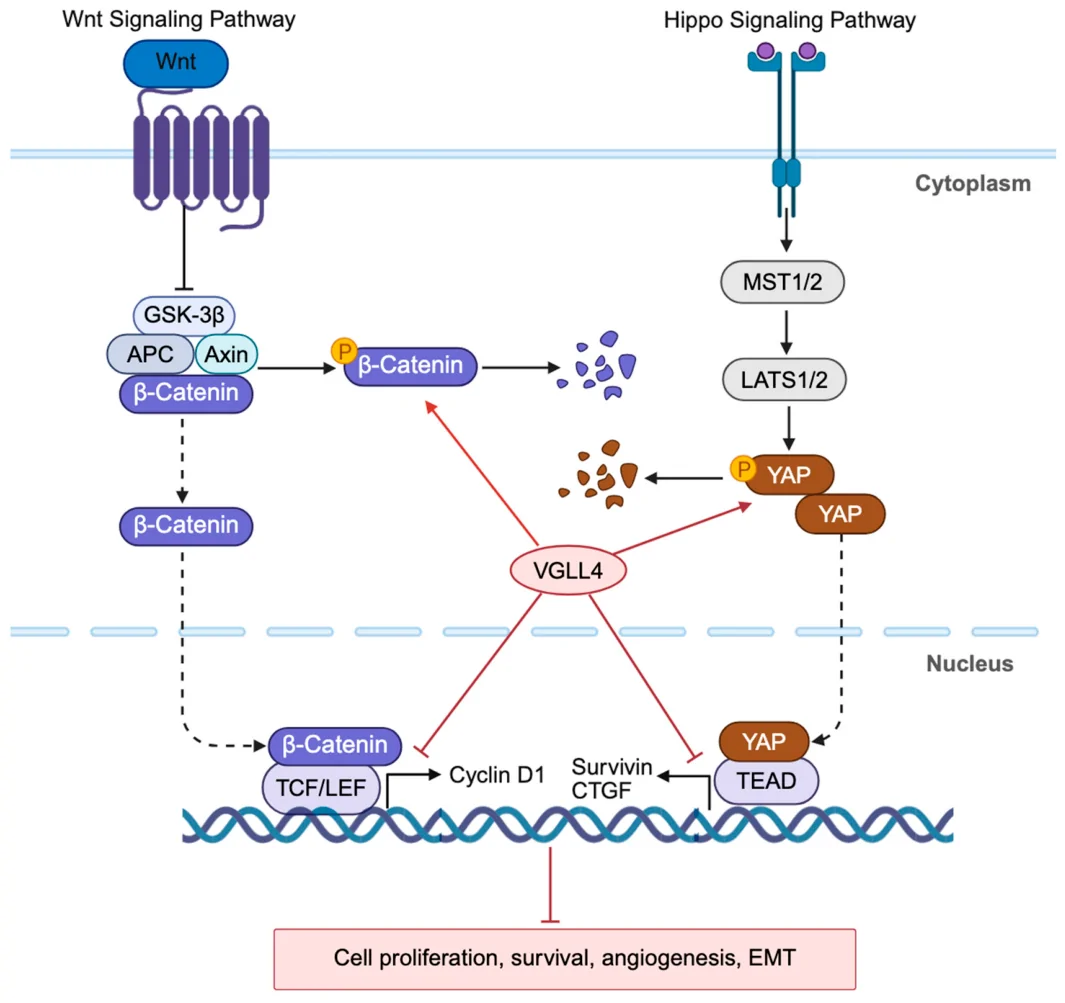
A model for the role of VGLL4 in suppressing ccRCC tumorigenesis via key signaling pathways (Created in BioRender. Yaru, Z. (2026) https://BioRender.com/3uorjpf (accessed on 5 June 2026)).

To further investigate whether the Hippo/YAP and Wnt/β-catenin pathways contribute to the tumor-suppressive effects induced by VGLL4 upregulation, we performed rescue experiments. We treated Caki-1-VGLL4 cells with GA-017 (a LATS1/2 inhibitor that can activate YAP) or CHIR-99021 (a GSK-3α/β inhibitor that can activate β-catenin) [23,24]. As shown in Figure 7E,F, both inhibitors effectively attenuated the VGLL4 overexpression-induced increase in phosphorylated YAP and phosphorylated β-catenin levels. Consequently, the impaired proliferative and clonogenic capacities of Caki-1-VGLL4 cells were partially but significantly restored upon pharmacological activation of either the YAP or β-catenin (Figure 7G,H). These results support that VGLL4 exerts its tumor-suppressive role in ccRCC at least in part by inhibiting both the Hippo/YAP and Wnt/β-catenin pathways, specifically through promoting YAP and β-catenin inactivation and restricting the transcriptional activity of YAP (Figure 8).

### 3.7. VGLL4 Suppresses Tumor Growth In Vivo

**Figure 9:**
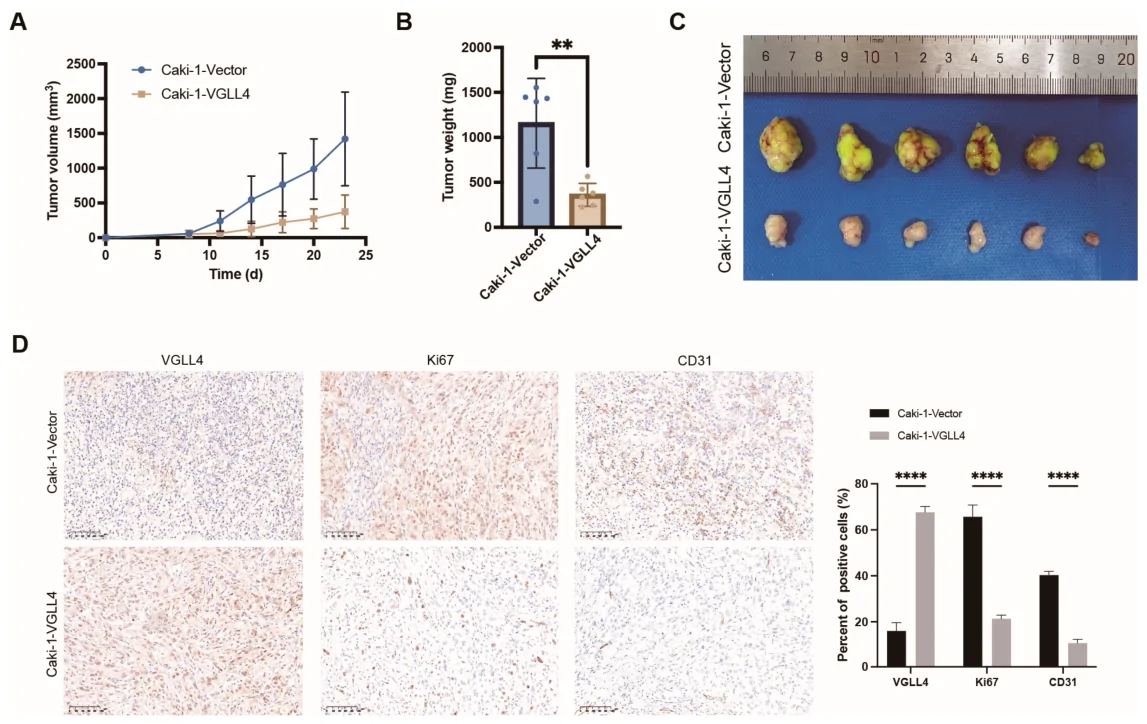
VGLL4 overexpression suppresses tumor growth of ccRCC cells in vivo. (**A**) Tumor growth curves were measured every three days after xenograft formation (*n* = 6). (**B**) Final tumor weight at the experimental endpoint (*n* = 6). (**C**) Representative images of excised tumors from each group. (**D**) Immunohistochemical staining of xenograft tissues for VGLL4, Ki67, and CD31 (*n* = 3). Scale bars represent 100 μm. Data are presented as the mean ± SD. ** *p* < 0.01 and **** *p* < 0.0001 indicate significant difference.

We established subcutaneous xenograft models using Caki-1-VGLL4 and Caki-1-Vector cells to further evaluate the tumor-suppressive role of VGLL4 in vivo. As exhibited in Figure 9A, VGLL4 overexpression remarkably delayed the growth of xenograft tumors compared to the vector group.

Consistently, both final tumor weight (Figure 9B) and representative tumor images (Figure 9C) demonstrated marked growth inhibition. At the endpoint of the experiment, the average tumor weight of the Caki-1-VGLL4 group was less than half that of the vector control group (Figure 9B). Immunohistochemical staining confirmed sustained high expression of VGLL4 protein in the Caki-1-VGLL4 xenografts (Figure 9D). Moreover, compared with the Caki-1-vector group, the expression of proliferation marker Ki67 and endothelial marker CD31 was all significantly decreased in the Caki-1-VGLL4 group (Figure 9D), indicating an anti-proliferation activity of VGLL4 in vivo. Collectively, these results demonstrate that VGLL4 also effectively suppresses ccRCC tumor growth in vivo.

## 4. Discussion

It is well established that the Hippo/YAP and Wnt/β-catenin signaling pathways play crucial roles in the initiation and progression of ccRCC. Aberrant activation of YAP has been frequently observed in ccRCC and is closely associated with enhanced tumor cell proliferation, angiogenesis, and poor clinical outcomes [25,26]. Similarly, dysregulated Wnt/β-catenin signaling, particularly characterized by cytoplasmic accumulation and nuclear translocation of β-catenin, correlates with advanced tumor stage, aggressive behavior, and unfavorable prognosis in ccRCC [27,28,29,30]. These pathways are therefore considered pivotal drivers of ccRCC malignancy. VGLL4 has emerged as an important transcriptional regulator involved in multiple signaling pathways, with its tumor-suppressive role most extensively characterized in the context of Hippo signaling. Mechanistically, VGLL4 functions as a transcriptional co-repressor by competitively binding TEAD transcription factors, thereby antagonizing YAP-mediated gene transcription [31]. Consistent tumor-suppressive effects of VGLL4 have been reported in several malignancies, including epidermal squamous cell carcinoma, breast cancer, gastric cancer, lung cancer, and hepatocellular carcinoma, where VGLL4 primarily exerts its function through disruption of the YAP-TEAD complex [8,11,13,32,33]. Beyond Hippo signaling, VGLL4 also participates in the regulation of Wnt/β-catenin signaling. In gastric cancer, VGLL4 has been shown to alter the nuclear localization of β-catenin and negatively regulate Wnt/β-catenin-dependent transcription, thereby suppressing epithelial–mesenchymal transition [34]. In colorectal cancer, VGLL4 was reported to target the TEAD4–TCF4 transcriptional complex, enabling coordinated regulation of both the Hippo/YAP and Wnt/β-catenin pathways [35]. These studies highlight VGLL4’s potential as a dual-pathway regulator.

Building upon these observations, the present study provides evidence that VGLL4 acts as a tumor suppressor in ccRCC. We observed that VGLL4 was significantly downregulated in ccRCC tissues, and its low expression correlated with unfavorable clinicopathological features and poorer overall survival, with a consistent but non-significant trend toward poorer disease-free survival after multivariate adjustment. Functionally, restoring VGLL4 expression inhibited ccRCC cell proliferation, migration, invasion, and tumor growth in vivo. Mechanistically, our findings suggest that VGLL4 exerts its effects at least in part through suppression of both Hippo/YAP and Wnt/β-catenin pathways in ccRCC. This is supported by the findings that VGLL4 upregulation increased the phosphorylation of both YAP and β-catenin, decreased the expression of their downstream targets (e.g., CTGF, Survivin, and Cyclin D1), and reversed EMT-related markers. Moreover, the tumor-suppressive phenotype was partially rescued by pharmacologically activating either pathway, confirming their functional relevance. The mechanistic findings of this study are highlighted by two main aspects. First, while VGLL4 has been reported as a tumor suppressor in several cancers, primarily through Hippo/YAP inhibition, our work provides experimental evidence in ccRCC that VGLL4 can suppress both the Hippo/YAP and Wnt/β-catenin pathways. To our knowledge, such co-suppression of these two oncogenic cascades by VGLL4 has been documented primarily in gastric and colorectal cancer [34,35]. These dual regulatory effects suggest that VGLL4 may act as a potential integrator of two major signaling axes in ccRCC, pointing to a potentially underappreciated layer of pathway crosstalk in this malignancy. Second, beyond the established model in which VGLL4 competes with YAP for TEAD binding to suppress YAP transcriptional activity, we found that VGLL4 upregulation also promotes YAP phosphorylation, thereby inhibiting its activation. This mechanism has rarely been reported in other tumor types and is described here in ccRCC.

Although our findings support a potential tumor-suppressive role of VGLL4 in ccRCC progression, several limitations should be acknowledged. First, von Hippel-Lindau (VHL) gene is an important tumor suppressor in ccRCC and its loss-of-function mutation serves as a hallmark of ccRCC [36,37]. In the present study, while we utilized a panel of ccRCC cell lines encompassing both VHL wild-type (Caki-1, ACHN) and VHL mutant (OS-RC-2) backgrounds to assess the general role of VGLL4, the experimental design did not systematically investigate whether VHL status modulates VGLL4 function or its interaction with the Hippo/YAP and Wnt/β-catenin pathways. Future studies employing isogenic VHL-knockout or -rescue models are warranted to dissect any potential crosstalk between the VHL/HIF axis and VGLL4-mediated signaling in ccRCC progression. Second, the precise mechanism by which VGLL4 enhances YAP/β-catenin phosphorylation remains unclear, which calls for further investigation into whether it affects kinases like LATS1/2 or components of the β-catenin destruction complex. Third, the lack of small-molecule modulators for VGLL4 poses a significant challenge for developing VGLL4-targeted anti-tumor therapies. In our preliminary screening, we observed that the PI3K/mTOR inhibitor BEZ235 could upregulate VGLL4 at the transcriptional level. However, due to its low selectivity toward VGLL4, it was not employed in the present study. Therefore, identifying highly selective and potent small-molecule modulators of VGLL4 remains a key focus for our future work.

In conclusion, this study suggests that VGLL4 functions as a tumor suppressor in ccRCC, at least in part by inhibiting both the Hippo/YAP and Wnt/β-catenin signaling cascades. These findings deepen the mechanistic insight into VGLL4’s role in ccRCC and underscore its potential as a prognosis-associated marker and candidate therapeutic target.

## Data Availability

The raw FASTQ files and processed data generated in this study have been deposited in the NCBI Gene Expression Omnibus (GEO) under accession number GSE 348814 (https://www.ncbi.nlm.nih.gov/geo/query/acc.cgi?acc=GSE348814 accessed on 29 September 2026). The analysis code used in this study is available at Zenodo under DOI 10.5281/zenodo.22942530 (https://doi.org/10.5281/zenodo.22942530 accessed on 29 September 2026).

## Supplementary Materials

The following supporting information can be downloaded at: https://www.mdpi.com/article/10.3390/biomedicines14102334/s1, Figure S1: VGLL4 protein expression in normal kidney tissue and ccRCC tissue (HPA database); Figure S2: Heatmap of DEGs enriched in GSEA terms following VGLL4 overexpression (DNA replication and activation of the pre-replicative complex); Table S1: Optimal cutoff of VGLL4 expression determined by X-tile; Table S2: Univariate and multivariate Cox regression analyses of overall survival (OS) in patients with ccRCC from the TCGA-KIRC cohort; Table S3: Univariate and multivariate Cox regression analyses of disease-free survival (DFS) in patients with ccRCC from the TCGA-KIRC cohort.

## References

[1] Bray F., Laversanne M., Sung H., Ferlay J., Siegel R.L., Soerjomataram I., Jemal A. (2024). Global cancer statistics 2022: GLOBOCAN estimates of incidence and mortality worldwide for 36 cancers in 185 countries. CA Cancer J. Clin..

[2] Mattila K.E., Vainio P., Jaakkola P.M. (2022). Prognostic Factors for Localized Clear Cell Renal Cell Carcinoma and Their Application in Adjuvant Therapy. Cancers.

[3] Gao R., Pang J., Lin P., Wen R., Wen D., Liang Y., Ma Z., Liang L., He Y., Yang H. (2024). Identification of clear cell renal cell carcinoma subtypes by integrating radiomics and transcriptomics. Heliyon.

[4] Deng J., Tu S., Li L., Li G., Zhang Y. (2024). Diagnostic, predictive and prognostic molecular biomarkers in clear cell renal cell carcinoma: A retrospective study. Cancer Rep..

[5] Bedke J., Gauler T., Grunwald V., Hegele A., Herrmann E., Hinz S., Janssen J., Schmitz S., Schostak M., Tesch H. (2017). Systemic therapy in metastatic renal cell carcinoma. World J. Urol..

[6] Meng L., Collier K.A., Wang P., Li Z., Monk P., Mortazavi A., Hu Z., Spakowicz D., Zheng L., Yang Y. (2023). Emerging Immunotherapy Approaches for Advanced Clear Cell Renal Cell Carcinoma. Cells.

[7] Feng X., Wang Z., Wang F., Lu T., Xu J., Ma X., Li J., He L., Zhang W., Li S. (2019). Dual function of VGLL4 in muscle regeneration. EMBO J..

[8] Zhang W., Gao Y., Li P., Shi Z., Guo T., Li F., Han X., Feng Y., Zheng C., Wang Z. (2014). VGLL4 functions as a new tumor suppressor in lung cancer by negatively regulating the YAP-TEAD transcriptional complex. Cell Res..

[9] Deng X., Fang L. (2018). VGLL4 is a transcriptional cofactor acting as a novel tumor suppressor via interacting with TEADs. Am. J. Cancer Res..

[10] Jiao S., Wang H., Shi Z., Dong A., Zhang W., Song X., He F., Wang Y., Zhang Z., Wang W. (2014). A peptide mimicking VGLL4 function acts as a YAP antagonist therapy against gastric cancer. Cancer Cell.

[11] Zhang Y., Shen H., Withers H.G., Yang N., Denson K.E., Mussell A.L., Truskinovsky A., Fan Q., Gelman I.H., Frangou C. (2017). VGLL4 Selectively Represses YAP-Dependent Gene Induction and Tumorigenic Phenotypes in Breast Cancer. Sci. Rep..

[12] Wei J., Cao Z., Li Q., Li X., Wang Q., Zhang Y., Zhang R., Wu X., Dai Q., Li X. (2025). Nuclear ubiquitination permits Hippo-YAP signal for liver development and tumorigenesis. Nat. Chem. Biol..

[13] Mickle M., Adhikary G., Shrestha S., Xu W., Eckert R.L. (2021). VGLL4 inhibits YAP1/TEAD signaling to suppress the epidermal squamous cell carcinoma cancer phenotype. Mol. Carcinog..

[14] Bevanda D., Racetin A., Kelam N., Filipovic N., Bevanda M., Rudan Dimlic M., Budimir J., Bevanda Glibo D., Bevanda I., Ramljak D. (2025). Expression Pattern of AIFM3, VGLL4, and WNT4 in Patients with Different Stages of Colorectal Cancer. Cancers.

[15] Wu A., Wu Q., Deng Y., Liu Y., Lu J., Liu L., Li X., Liao C., Zhao B., Song H. (2019). Loss of VGLL4 suppresses tumor PD-L1 expression and immune evasion. EMBO J..

[16] Zhang Y., Wang X. (2020). Targeting the Wnt/beta-catenin signaling pathway in cancer. J. Hematol. Oncol..

[17] Song P., Gao Z., Bao Y., Chen L., Huang Y., Liu Y., Dong Q., Wei X. (2024). Wnt/beta-catenin signaling pathway in carcinogenesis and cancer therapy. J. Hematol. Oncol..

[18] Kiang K.M., Ahad L., Zhong X., Lu Q.R. (2024). Biomolecular condensates: Hubs of Hippo-YAP/TAZ signaling in cancer. Trends Cell Biol..

[19] Li H.L., Li Q.Y., Jin M.J., Lu C.F., Mu Z.Y., Xu W.Y., Song J., Zhang Y., Zhang S.Y. (2021). A review: Hippo signaling pathway promotes tumor invasion and metastasis by regulating target gene expression. J. Cancer Res. Clin. Oncol..

[20] Zhao H., Ming T., Tang S., Ren S., Yang H., Liu M., Tao Q., Xu H. (2022). Wnt signaling in colorectal cancer: Pathogenic role and therapeutic target. Mol. Cancer.

[21] Alsaab H.O. (2023). Pathological role of long non-coding (lnc) RNA in the regulation of Wnt/beta-catenin signaling pathway during epithelial-mesenchymal transition (EMT). Pathol. Res. Pract..

[22] Akrida I., Bravou V., Papadaki H. (2022). The deadly cross-talk between Hippo pathway and epithelial-mesenchymal transition (EMT) in cancer. Mol. Biol. Rep..

[23] Liang J., Guo S., Wang Y., Cao Q., Guo T., Zhou D., Li J., Liao Y., Zhong B., Jiang N. (2025). LINC00525 drives aggressive phenotypes in bladder cancer via YAP stabilization-mediated transcriptional activation. Cancer Cell Int..

[24] Ding D., Wang X., Xuan R., Li R., Zhang Y., Wang Z. (2026). Ephrin-B2 promotes gastric cancer growth by inhibiting apoptosis and regulating the cell cycle via the Wnt/beta-catenin signaling pathway. Int. J. Oncol..

[25] Lv X., Liu J., Islam K., Ruan J., He C., Chen P., Huang C., Wang H., Dhar A., Moness M. (2025). Hyperactivated YAP1 is essential for sustainable progression of renal clear cell carcinoma. Oncogene.

[26] Cao J.J., Zhao X.M., Wang D.L., Chen K.H., Sheng X., Li W.B., Li M.C., Liu W.J., He J. (2014). YAP is overexpressed in clear cell renal cell carcinoma and its knockdown reduces cell proliferation and induces cell cycle arrest and apoptosis. Oncol. Rep..

[27] Rozsypalova A., Rosova B., Filipova A., Nikolov D.H., Chloupkova R., Richter I., Zachoval R., Matej R., Melichar B., Buchler T. (2025). Association of the combined parameters including the frequency of primary cilia, PD-L1, Smoothened protein, membranous beta-catenin and cytoplasmic beta-catenin expression with the outcome of patients with clear cell renal cell carcinoma. BioMed Pap. Med. Fac. Univ. Palacky. Olomouc Czech Repub..

[28] Von Schulz-Hausmann S.A., Schmeel L.C., Schmeel F.C., Schmidt-Wolf I.G. (2014). Targeting the Wnt/beta-catenin pathway in renal cell carcinoma. Anticancer Res..

[29] Gorka J., Marona P., Kwapisz O., Waligorska A., Pospiech E., Dobrucki J.W., Rys J., Jura J., Miekus K. (2021). MCPIP1 inhibits Wnt/beta-catenin signaling pathway activity and modulates epithelial-mesenchymal transition during clear cell renal cell carcinoma progression by targeting miRNAs. Oncogene.

[30] Cai J., Huang Z., Zhou J., Wu W., Ye Y. (2022). TLN2 functions as a tumor suppressor in clear cell renal cell carcinoma via inactivation of the Wnt/beta-catenin signaling pathway. Transl. Androl. Urol..

[31] Cai J., Choi K., Li H., Pulgar Prieto K.D., Zheng Y., Pan D. (2022). YAP-VGLL4 antagonism defines the major physiological function of the Hippo signaling effector YAP. Genes Dev..

[32] Li N., Yu N., Wang J., Xi H., Lu W., Xu H., Deng M., Zheng G., Liu H. (2015). miR-222/VGLL4/YAP-TEAD1 regulatory loop promotes proliferation and invasion of gastric cancer cells. Am. J. Cancer Res..

[33] Feng X., Lu T., Li J., Yang R., Hu L., Ye Y., Mao F., He L., Xu J., Wang Z. (2020). The Tumor Suppressor Interferon Regulatory Factor 2 Binding Protein 2 Regulates Hippo Pathway in Liver Cancer by a Feedback Loop in Mice. Hepatology.

[34] Li H., Wang Z., Zhang W., Qian K., Liao G., Xu W., Zhang S. (2015). VGLL4 inhibits EMT in part through suppressing Wnt/beta-catenin signaling pathway in gastric cancer. Med. Oncol..

[35] Jiao S., Li C., Hao Q., Miao H., Zhang L., Li L., Zhou Z. (2017). VGLL4 targets a TCF4-TEAD4 complex to coregulate Wnt and Hippo signalling in colorectal cancer. Nat. Commun..

[36] Sato Y., Yoshizato T., Shiraishi Y., Maekawa S., Okuno Y., Kamura T., Shimamura T., Sato-Otsubo A., Nagae G., Suzuki H. (2013). Integrated molecular analysis of clear-cell renal cell carcinoma. Nat. Genet.

[37] Hu J., Tan P., Ishihara M., Bayley N.A., Schokrpur S., Reynoso J.G., Zhang Y., Lim R.J., Dumitras C., Yang L. (2023). Tumor heterogeneity in VHL drives metastasis in clear cell renal cell carcinoma. Signal Transduct. Target. Ther..

