# Supplementary material for: VGLL4 Suppresses Malignant Phenotypes of Clear Cell Renal Cell Carcinoma Cells Through Modulation of Hippo/YAP and Wnt/β-Catenin Signaling Pathways
Source: Biomedicines. 2026 Oct 14;14(10):2334. doi: 10.3390/biomedicines14102334 (PMC13701626; doi:10.3390/biomedicines14102334)
Supplement: Supplementary file 1 [file biomedicines-14-02334-s001.zip › biomedicines-4540013-supplementary.pdf]

Supplemental Materials

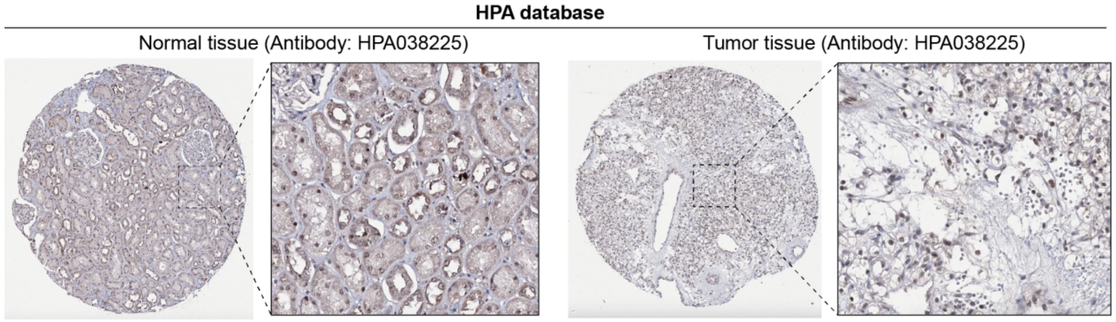

Figure S1. VGLL4 protein expression in normal kidney tissue and ccRCC tissue (HPA database).

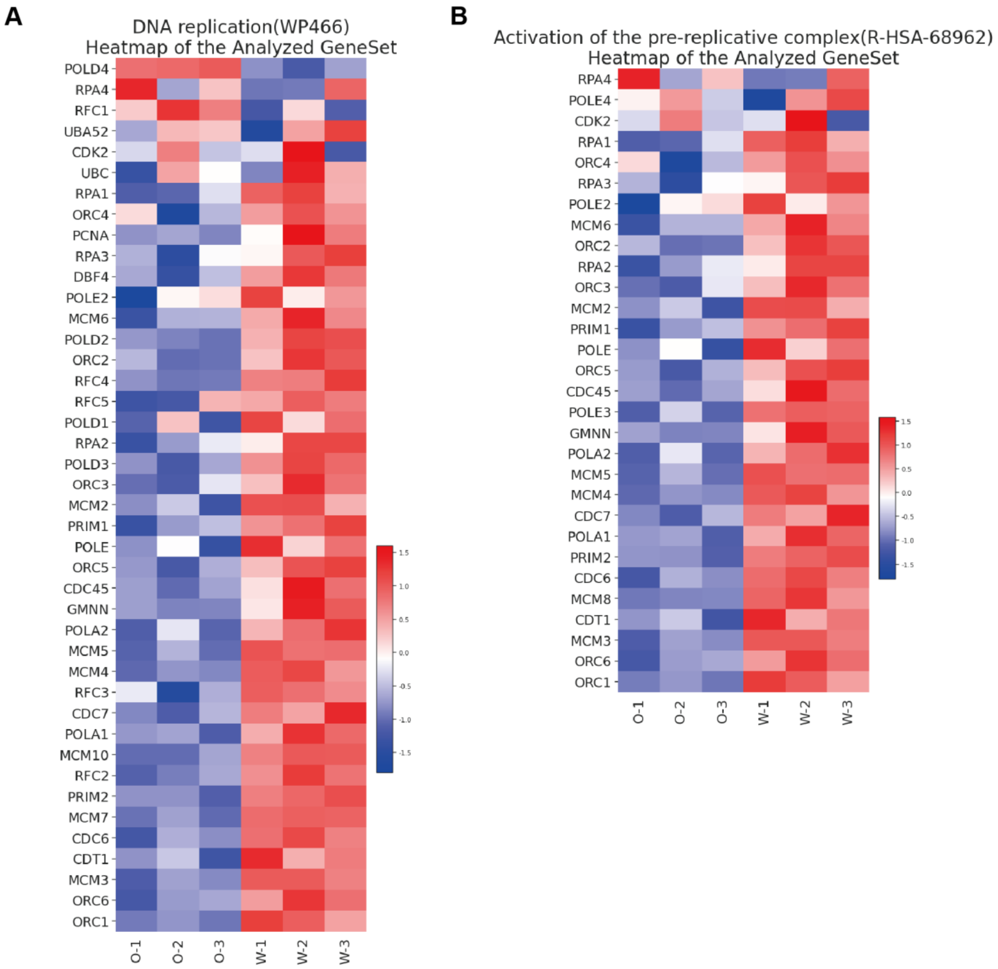

Figure S2. Heatmap of DEGs enriched in GSEA terms following VGLL4 overexpression (DNA replication and activation of the pre-replicative complex).

Item	Value
Software	X-tile 3.6.1
Marker	VGLL4 expression (TPM, primary tumor)
Endpoint	Overall survival
Patients	518
Optimal cutoff (TPM)	15.9
Chi-square (Hi/Lo)	18.5032
Miller-Siegmund P	0.0008
Relative risk (Low vs High)	2.02

Table S1. Optimal cutoff of VGLL4 expression determined by X-tile.

Variable	Comparison	Univariate HR (95% CI)	P value	Multivariate HR (95% CI)	P value
VGLL4 expression	High vs Low	0.50 (0.36–0.69)	<0.001	0.63 (0.45–0.87)	0.006
Age (years)	>60 vs ≤60	1.75 (1.28–2.38)	<0.001	1.42 (1.03–1.95)	0.031
Sex	Male vs Female	0.95 (0.70–1.30)	0.772	0.89 (0.65–1.23)	0.477
Pathologic stage	III–IV vs I–II	3.82 (2.77–5.26)	<0.001	2.91 (2.07–4.07)	<0.001
Histologic grade	G3–G4 vs G1–G2	2.66 (1.88–3.74)	<0.001	1.81 (1.26–2.59)	0.001

HR, hazard ratio; CI, confidence interval. n = 518, 171 deaths.

The multivariate model included VGLL4 expression, age, sex, pathologic stage and histologic grade.

VGLL4 expression (TPM, primary tumor) was dichotomized at 15.9, the optimal cutoff determined by X-tile 3.6.1.

Data source: TCGA-KIRC RNA-seq (STAR-Counts, TPM) and clinical data from the GDC.

Table S2. Univariate and multivariate Cox regression analyses of overall survival (OS) in patients with ccRCC from the TCGA-KIRC cohort.

Variable	Comparison	Univariate HR (95% CI)	P value	Multivariate HR (95% CI)	P value
VGLL4 expression	High vs Low	0.57 (0.39–0.82)	0.002	0.72 (0.49–1.05)	0.089
Age (years)	>60 vs ≤60	1.37 (0.96–1.95)	0.084	1.10 (0.76–1.59)	0.619
Sex	Male vs Female	1.43 (0.96–2.12)	0.077	1.29 (0.86–1.94)	0.225
Pathologic stage	III–IV vs I–II	6.53 (4.38–9.74)	<0.001	5.16 (3.42–7.79)	<0.001
Histologic grade	G3–G4 vs G1–G2	3.36 (2.22–5.07)	<0.001	2.15 (1.41–3.28)	<0.001

HR, hazard ratio; CI, confidence interval. n = 422, 123 recurrence/progression events.

The multivariate model included VGLL4 expression, age, sex, pathologic stage and histologic grade.

VGLL4 expression (TPM, primary tumor) was dichotomized at 15.9, the optimal cutoff determined by X-tile 3.6.1.

Data source: TCGA-KIRC RNA-seq (STAR-Counts, TPM) and clinical data from the GDC; disease-free survival from TCGA Firehose Legacy (cBioPortal).

Table S3. Univariate and multivariate Cox regression analyses of disease-free survival (DFS) in patients with ccRCC from the TCGA-KIRC cohort.
